# Supplementary material for: Delay in healthcare seeking for young children with severe pneumonia at Mulago National Referral Hospital, Uganda: A mixed methods cross-sectional study
Source: PLoS One. 2023 Oct 10;18(10):e0291387. doi: 10.1371/journal.pone.0291387 (PMC10564236; doi:10.1371/journal.pone.0291387)
Supplement: Attachment — Submitted filename: Response to Reviewers.docx [file pone.0291387.s001.docx]

10^th^ May, 2023

Academic Editor,

PLOS ONE

Dear Editor,

**Re: Response to reviewers’ comments on our manuscript titled: Delay in healthcare seeking for young children with severe pneumonia at Mulago National Referral Hospital, Uganda: a mixed methods cross-sectional study. (Ref No:** **PONE-D-22-33296)**

We are grateful for the comments and suggestions on our manuscript by the reviewers. We have addressed them and specified our response to each of the reviewers’ comments.

**Reviewer #1:**

**Comment:** Revise the sampling procedure, it say convenient method for the quantitative.

**Response:** We thank the reviewer for highlighting this correction. The sampling method has been revised to state that the consecutive method of sampling was used instead of convenience method **(See page 4, line 129).** The children were then enrolled consecutively until the desired sample size was realized. Studies on sampling methods have indicated that consecutive sampling is feasible and offers representative samples in clinical studies (1). So, the assumption was that the children that were sampled were representative of those that are seen at the emergency paediatric unit of Mulago hospital.

**Comment:** For the qualitative they used four FGDs why four?

**Response:** Four FGDs were carried out at the end of which saturation (2) was reached where no new insights were emerging. This clarification has been made **(See page 4, lines 124 – 126).**

**Comment:** In the analysis part, it is better to run both Bivariable and multivariable

**Response:** The analysis section has been elaborated extensively and tables updated to describe the multilevel modeling done detailing both bivariable and multivariable analysis. A logistic regression model was built by including all factors with p<0.2 at bivariable analysis for multivariable analysis. p≤0.05 at multivariable analysis was considered statistically significant. All statistical tests were 2-sided and considered statistically significant at α = 0.05. Odds ratios, 95% confidence intervals (CI) and p values are presented. Interaction was assessed using likelihood ratios comparing full and reduced models whereas confounding was assessed by comparing adjusted and unadjusted odds ratios and a predictor variable with a difference of ≥10% was considered as a confounder. Conclusions were drawn based on the adjusted OR with their corresponding 95% confidence intervals. Goodness of fit of the final model was assessed using the Hosmer-Lemeshow test. **(See page 7, lines 187 – 202, table 5 and table 6)**

**Reviewer #2**: In its current form, this manuscript is not publishable. More specific:
**Comment:** 1. Introduction is not specific and very long

**Response:** We thank the reviewer for highlighting this concern. The introduction section has extensively been revised, shortened, and made more focused. It has been re-written to focus on the burden of pneumonia, delay in seeking care and associated factors. In the initial version of the manuscript, it was 2.5 pages and has now been reduced to 1.5 pages. **(See pages i- ii, lines 55-93)**
**Comment:** 2. Methods are not described appropriately, including how sample size was calculated

**Response:** Thank you for this comment. We have revised the methods section entirely and also made explicit how the sample size was calculated**.** The sample size to determine the prevalence of delay in seeking healthcare was estimated using the formula for single proportions. We assumed 95% level of confidence, 5% sampling error, proportion of caregivers that delayed seeking healthcare for their children of 47.7% (3). This gave an estimated sample size of 384. In addition, we estimated the sample size for determining factors associated with delay in seeking healthcare based on the formular for comparison of two proportions using use of self-medication as the exposure variable (4). We assumed 5% significance, 80% power of the study, proportion of caregivers who had used self-medication was 25.6% versus 74.4% that had not used self-medication. The proportion of caregivers that had used self-medication and had delayed seeking healthcare was estimated at 78.7% while those that did not use self-medication but delayed was 42.1%. Based on these parameters, the estimated sample size was 66. The higher sample size of 384 based on the modified Kish Leslie formula was considered appropriate for the study **(See page 3 - 4, lines 111-127).**
**Comment:** 3. Analysis was not performed appropriately. More appropriate to conduct multilevel modelling

**Response:** More details on analysis have been added in the revised manuscript. Specifically, details on how multilevel modeling was done have been made explicit and tables updated (tables 5 and 6).

Descriptive statistics were used to analyse the participant characteristics and results expressed as proportions, means and medians, as appropriate. Shapiro wilks test was used to determine skewness of the different variables and the continuous variables that were not normally distributed were expressed as medians. To determine the prevalence of delay in seeking healthcare, the number of caregivers who took their children to a health center or hospital beyond 24 hours of onset of symptoms, was divided by the total number of participants and the result was expressed as a proportion. To determine the factors associated with delay in seeking healthcare, multivariable analysis was undertaken. A logistic regression model was built by including all factors with p<0.2 at bivariate analysis for multivariable analysis. P≤0.05 at multivariable analysis was considered statistically significant. All statistical tests were 2-sided and considered statistically significant at α = 0.05. Odds ratios, 95% confidence intervals (CI) and p values are presented. Interaction was assessed using likelihood ratios comparing full and reduced models whereas confounding was assessed by comparing adjusted and unadjusted odds ratios and a predictor variable with a difference of ≥10% was considered as a confounder. Conclusions were drawn based on the adjusted OR with their corresponding 95% confidence intervals. Goodness of fit of the final model was assessed using the Hosmer-Lemeshow test.

The qualitative data was analysed using content thematic approach in line with the study objectives. Transcripts were read several times to identify themes and sub-themes. Coding was done manually by PE in consultation with JR. This was done by grouping data in line with the themes and sub-themes. Verbatim quotations reflecting participants’ views on barriers and facilitators for prompt care seeking for children with severe pneumonia were identified and have been used in the presentation of the study findings. **(See pages 7- 8, lines 204 - 226)
Comment:** 4. Discussion section is not very well written

**Response:** We thank the reviewer for this comment. We have revised and strengthened the discussion section highlighting the key results, what they mean and compared with other studies. **(See pages 29 - 34)**

1.	Bjørn M, Brendstrup C, Karlsen S, Carlsen JE. Consecutive screening and enrollment in clinical trials: the way to representative patient samples? Journal of cardiac failure. 1998;4(3):225-30.

2.	Hennink MM, Kaiser BN, Marconi VC. Code saturation versus meaning saturation: how many interviews are enough? Qualitative health research. 2017;27(4):591-608.

3.	Källander K, Hildenwall H, Waiswa P, Galiwango E, Peterson S, Pariyo G. Delayed care seeking for fatal pneumonia in children aged under five years in Uganda: a case-series study. Bulletin of the World Health Organization. 2008;86:332-8.

4.	Bantie GM, Meseret Z, Bedimo M, Bitew A. The prevalence and root causes of delay in seeking healthcare among mothers of under five children with pneumonia in hospitals of Bahir Dar city, North West Ethiopia. BMC pediatrics. 2019;19(1):482.
