# Supplementary material for: Delay in healthcare seeking for young children with severe pneumonia at Mulago National Referral Hospital, Uganda: A mixed methods cross-sectional study
Source: PLoS One. 2023 Oct 10;18(10):e0291387. doi: 10.1371/journal.pone.0291387 (PMC10564236; doi:10.1371/journal.pone.0291387)
Supplement: Attachment — Submitted filename: Response to Reviewers.docx [file pone.0291387.s002.docx]

August 8, 2023

Academic Editor,

PLOS ONE

Dear Editor,

**Re: Response to the editors’ comments on our manuscript titled: Delay in healthcare seeking for young children with severe pneumonia at Mulago National Referral Hospital, Uganda: a mixed methods cross-sectional Study. (Ref No:** **PONE-D-22-33296R1)**

We appreciate the comments on our manuscript. We have addressed them and specified our response to each of the comments.

**Comment:** Please review your reference list to ensure that it is complete and correct. If you have cited papers that have been retracted, please include the rationale for doing so in the manuscript text, or remove these references and replace them with relevant current references. Any changes to the reference list should be mentioned in the rebuttal letter that accompanies your revised manuscript. If you need to cite a retracted article, indicate the article’s retracted status in the References list and also include a citation and full reference for the retraction notice.

**Response:** We thank the reviewer for highlighting this correction. We have reviewed the reference list and corrected any misspelled words. We are sure the list is complete and correct. No cited papers have been retracted.

**Comment:** Please check the spelling and grammatical errors.

**Response:** We have read the whole manuscript and corrected all the spelling and grammatical errors as marked up in the track changes**.**

**Comment:** Also, revise the tables with row percentage (For example: if you are comparing delay and not delay with other variables). Thank you.

**Response:** The tables with row percentages have been revised to have percentages of caregivers with a variable who delayed versus those with the same variable who did not delay. **(See page 15 and 17, table 5 and table 6)**
